# Individual risk factors for *Mycoplasma hyopneumoniae* infections in suckling pigs at the age of weaning

**DOI:** 10.1186/1751-0147-55-44

**Published:** 2013-06-03

**Authors:** Heiko Nathues, Stefanie Doehring, Henrike Woeste, Anna S Fahrion, Marcus G Doherr, Elisabeth grosse Beilage

**Affiliations:** 1 Field Station for Epidemiology, University of Veterinary Medicine Hannover, Foundation, Buescheler Street 9, Bakum,, D-49456, Germany; 2 Veterinary Public Health Institute, Department of Clinical Research - Veterinary Public Health, Vetsuisse Faculty, University of Bern, Schwarzenburgstrasse 155, Liebefeld (BE), CH-3097, Switzerland; 3 Department of Production and Population Health, Veterinary Epidemiology, Economics and Public Health Group, Hawkshead Lane, North Mymms, Hatfield, Hertfordshire, AL9 7TA, United Kingdom

**Keywords:** *Mycoplasma hyopneumoniae*, Enzootic pneumonia, Suckling pig, Epidemiology, Risk factor analysis

## Abstract

**Background:**

In recent years, the occurrence and the relevance of *Mycoplasma hyopneumoniae* infections in suckling pigs has been examined in several studies. Whereas most of these studies were focused on sole prevalence estimation within different age groups, follow-up of infected piglets or assessment of pathological findings, none of the studies included a detailed analysis of individual and environmental risk factors. Therefore, the aim of the present study was to investigate the frequency of *M. hyopneumoniae* infections in suckling pigs of endemically infected herds and to identify individual risk factors potentially influencing the infection status of suckling pigs at the age of weaning.

**Results:**

The animal level prevalence of *M. hyopneumoniae* infections in suckling pigs examined in three conventional pig breeding herds was 3.6% (41/1127) at the time of weaning. A prevalence of 1.2% was found in the same pigs at the end of their nursery period. In a multivariable Poisson regression model it was found that incidence rate ratios (IRR) for suckling pigs are significantly lower than 1 when teeth grinding was conducted (IRR: 0.10). Moreover, high temperatures in the piglet nest during the first two weeks of life (occasionally >40°C) were associated with a decrease of the probability of an infection (IRR: 0.23-0.40). Contrary, the application of PCV2 vaccines to piglets was associated with an increased infection risk (IRR: 9.72).

**Conclusions:**

Since single infected piglets are supposed to act as initiators for the transmission of this pathogen in nursery and fattening pigs, the elimination of the risk factors described in this study should help to reduce the incidence rate of *M. hyopneumoniae* infections and thereby might contribute to a reduced probability of high prevalences in older pigs.

## Background

*Mycoplasma hyopneumoniae*, the etiologic agent of enzootic pneumonia in pigs, is widespread in most countries worldwide. Both the infection and the disease play a prominent role in the porcine respiratory disease complex (PRDC), which is usually affecting pigs aged 16 to 20 weeks [1]. In general, it is accepted that the occurrence, course and severity of enzootic pneumonia is influenced by a number of factors such as virulence of the particular strain [2] as well as the additional occurrence of other respiratory pathogens and miscellaneous risk factors [1].

*M. hyopneumoniae* is mainly transmitted horizontally from infected pigs to non-infected pen mates, but is also transmitted vertically from sows to their offspring by the frequent and close nose-to-nose contact during the suckling period [3]. The relevance of the latter way of transmission is not questioned, since infected piglets are considered as initiators for the spread of the pathogen during the following production stages [4-6]. Nonetheless, detailed knowledge about individual risk factors for suckling pigs is lacking, although the identification of risk factors in individual herds, mainly comprised by husbandry and management factors, and their reduction is a prerequisite for disease control and prevention.

In several studies numerous risk factors for the infection of growing and fattening pigs with *M. hyopneumoniae* have been examined [7-11]. However, only few studies focused on the potentially very important role of suckling and nursery pigs and their individual risk factors for positivity to *M. hyopneumoniae.* These studies were focused on prevalence within different age groups [12], follow-up of infected piglets [6] or pathological findings [13], whereas none of the studies included a detailed analysis of individual and environmental risk factors. Moreover, prevalences of *M. hyopneumoniae* in suckling and nursery pigs assessed in former studies are inconsistent and vary from 1.5% [14] to 58% [12].

The aim of the present study was to estimate the frequency of *M. hyopneumoniae* infections in suckling pigs and to identify individual risk factors potentially influencing the infection status of suckling pigs at the age of weaning. Finally, the consequence of positivity in suckling pigs for the spread of the infection in the nursery unit was estimated.

## Methods

An observational cross-sectional study was conducted in three pig breeding herds between December 2009 and June 2010. The study was performed in compliance with the guidelines for ‘Good Clinical Practise’ (GCP) [15] under licence for experimenting on animals from the German Federal State 81 Veterinary Administration Offices in Lower Saxony (No. 33.9-42502-05-11A104; LAVES, Oldenburg, Germany).

### Selection of herds

For this study three pig herds in the north-western part of Germany, where at least three out of 20 suckling pigs had been tested positive for *M. hyopneumoniae* by PCR were selected (Table 1). All herds were located in the north-western part of Germany, which is characterised by a high pig density of more than 800 pigs / km^2^. The inclusion criteria defined were that herds had to be kept on a one-site or two-site production system making sure that sows, suckling pigs and nursery pigs were available for examination, and a minimum herd size of 120 producing sows. Furthermore, the farrowing units and the nursery units had to be located in the same place. The vaccination of the sows against *M. hyopneumoniae* was an exclusion criterion. All herds were housing pigs in conventional husbandry systems.

**Table 1:** General characteristics of the study herds

**Herd No.**	**Sows (n)**	**Nursery pigs (n)**	**Fattening pigs (n)**	**Production rhythm (week)**
1	180	750	0	1*
2	500	1,700	550	3
3	500	2,500	3,500	2

*with variation in batch size.

### Selection of animals

In each of the three herds 45 sows and their offspring were selected for this study. A random stratified selection according to the individual number of parities of the sows was performed considering the age structure of the particular herds. Fifteen sows from three subsequent farrowing batches suitable for the purpose of this study were enrolled three weeks prior to their estimated farrowing date.

### Parameters registered on animal level

Apart from the sampling of the sows three weeks ante partum and the final sampling of the piglets towards the end of the nursery phase, all animals were examined on a daily basis from farrowing / birth until weaning. Clinical examination and data recording were always conducted by the same investigator.

In sows, the following parameters were recorded:

▪ Date and time of farrowing (during normal working hours vs. out of working hours) and subsequent behaviour (e.g. aggression)

▪ Number of total born, live born, dead born and weaned piglets of the current litter, as well as overall number of parities

▪ Total number of teats and number of functional teats (counted as ‘pairs of teats from cranial to caudal’)

▪ Occurrence of systemic disease (e.g. post-partum dysgalactia syndrome (PPDS), etc.) and/ or local disease (e.g. arthritis) and the corresponding facultative treatments applied to single animals (time, substance, etc.).

Treatments with amoxicillin, ampicillin, colistin and penicillin were considered being ‘not effective against *M. hyopneumoniae*’. In contrast to this, all remaining antimicrobials that were used for the treatment of animals enrolled in this study were taken into account as being ‘effective against *M. hyopneumoniae*’. These antimicrobials namely were apramycin, enrofloxacin, tetracyclin and tulathromycin.

▪ Duration of suckling period and number of cross-fostered piglets

▪ Level of serum antibodies against *M. hyopneumoniae* approx. three weeks ante partum and approx. 12 to 60 hours after farrowing (0.5 to 2.5 days)

▪ Occurrence of *M. hyopneumoniae* in nasal swabs approx. 12 to 60 hours after farrowing (0.5 to 2.5 days) and at weaning

In piglets, the following parameters were recorded:

▪ Day of birth and gender

▪ Body weight subsequently after birth (weighing was done on the same day, when farrowing was during the day or next day, when farrowing was during the night) and health status including congenital abnormalities

The body weight of the piglets was measured using a high resolution platform scale (FG 15OK AK, A&D Instruments LTD, Ahrensburg, Germany).

▪ Preferred teat for suckling (estimation based on five different observations)

▪ Occurrence of systemic and/ or local disease (e.g. diarrhoea or arthritis) and the corresponding facultative treatments applied to single animals (time, substance, etc.) Treatments were assorted as ‘effective’ or ‘not effective’ against *M. hyopneumoniae* (see sows)

▪ Time of routine procedures: castration, teeth grinding, tail docking, iron application, ear tagging, potential cross-fostering, etc.

▪ Time of vaccination including product and dosage

▪ Routine treatment with antimicrobials applied to all piglets (time, substance, dosage, etc.)

▪ Level of serum antibodies against *M. hyopneumoniae* at 14 days of age in order to determine the uptake of maternally derived antibodies

▪ Occurrence of *M. hyopneumoniae* in nasal swabs at weaning

▪ Day of weaning and body weight at that time

### Parameters registered on pen level and in the environment

In the farrowing unit, the average temperature in the piglet nest was determined at the day of birth and subsequently at day 7, 14 and 21 post natum. All measurements were conducted using an infrared thermometer (Voltcraft® IR 650-12D, Conrad Electronic SE, Hirschau, Germany) and were run in triplicates. The room temperature and the temperature outside of the barn were recorded continuously using data-logger (175-T1 Logger, Testo AG, Lenzkirch, Germany). The minimum, maximum and average temperatures for each day were entered into the data base.

In the nursery unit, the size of the pens, air volume of compartments and the number of pigs per pen was assessed.

### Collection of samples

#### Nasal swabs

**Figure 1:**
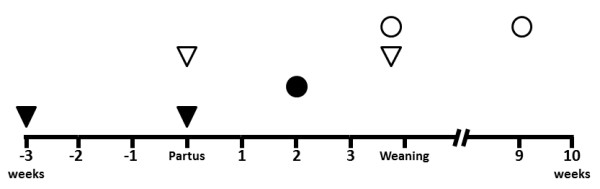
**Time schedule of sample collection from sows (triangles) and offspring (circles).** Filled symbols represent blood samples and unfilled symbols represent nasal swabs.

Nasal swabs were collected from all sows within 60 hours after farrowing and at the end of the suckling period, which lasted 24.8 days on average (Figure 1). Furthermore, nasal swabs from all piglets were taken first at the time of weaning and second towards the end of the nursery period at approximately 9 weeks of age (Figure 1). For each sample, the examiner changed the disposable gloves, and the nose of each pig was dry-cleaned with a disposable paper in order to prevent any contamination. Subsequently, the swabs (Dacron-swab, MAST Diagnostics Group Ltd., Reinfeld, Germany) were consecutively inserted into the ventral passages of both nostrils. Swabs were pushed forward and remained at least 3 seconds in this position.

#### Blood samples

##### From sows

Blood samples were collected from sows before farrowing (3 weeks *ante-partum*) and within 12–60 hours after farrowing (*post-partum*) for the purpose of estimating the transfer of maternal antibodies against *M. hyopneumoniae* with the colostrum (Figure 1). It was assumed that this is correlated with the decrease of the concentration of serum antibodies.

##### From suckling pigs

Blood samples were collected at 2 weeks of age in order to determine the concentration of maternally derived antibodies against *M. hyopneumoniae* (Figure 1).

### Detection of *M. hyopneumoniae* by real-time PCR

DNA isolation from nasal swabs was always conducted at the day of sampling. The top of each swab was clipped and incubated in 1.5 ml sterilized Tris-EDTA buffer for 30 min at 56°C. After transferring the top of the swab into a shortened filter tip, which was placed in a new reaction tube, this tube was centrifuged at 18,000 g for 15 sec. Subsequently, the shortened filter tip containing the swab was discarded, while the liquid on the bottom of the reaction tube was transferred into the corresponding reaction tube, which contained the Tris-EDTA buffer from the first incubation. The samples were centrifuged at 18,000 g lasting 20 min. After discarding the liquid, pellets were submitted to DNA isolation using a silica-membrane-based spin kit according to the manufacturer’s instructions (QIAamp DNA Mini kit, Qiagen). Amplification of DNA was performed using a multiplex real-time PCR [16,17] on an AB 7500 system (Life-technologies).

### Detection of antibodies against *M. hyopneumoniae* by ELISA

Blood samples were kept at room temperature for 2 to 3 hours in order to guarantee sufficient time for clotting. Subsequently, they were centrifuged at 2,000 g lasting 10 min and serum was transferred in 1.5 ml reaction tubes. To avoid an examination with different lots/batches of reagents and ELISA plates, serum was stored at −20°C until all samples of the three herds were collected. The serum was examined for antibodies against *M. hyopneumoniae* using an ELISA according to the manufacturer’s instructions (HerdCheck® M. hyo, Idexx Laboratories).

The sample/positive-ratios (S/P-ratios) were calculated based on optical densities (OD) as follows:

$$S/P=\frac{{\mathrm{OD}}_{\mathrm{sample}}{-\mathrm{OD}}_{\mathrm{mean}\;\mathrm{negative}\;\mathrm{control}}}{{\mathrm{OD}}_{\mathrm{mean}\;\mathrm{positive}\;\mathrm{control}}{-\mathrm{OD}}_{\mathrm{mean}\;\mathrm{negative}\;\mathrm{control}}}$$

### Data handling and statistical analysis

Data were collected using accordingly structured and standardised data collection forms in compliance with the guidelines for ‘Good Clinical Practise’ (GCP) [15]. Separate forms were used to store data related to the sows, the litters, the piglets and the environment.

Observational data from the three herds as well as results from laboratory testing were entered into a database (Microsoft Office Access 2010, http://www.microsoft.com). After transferring and merging all data into a spread-sheet program (Microsoft Office Excel 2010) data was analysed using NCSS version 07.1.4 (http://www.NCSS.com) and Stata IC 12 (http://www.stata.com).

The individual piglet was defined as the statistical unit and the corresponding PCR result at the time of weaning – determining ‘infection with *M. hyopneumoniae*‘ versus ‘no infection with *M. hyopneumoniae*’ - was assigned being the outcome variable (dependent variable). Since a positive outcome was rare, a Poisson-type regression model was considered most appropriate to assess the association with potential risk factors (independent variables) influencing the infections status of piglets at the time of weaning. The outcome reflects the infection risk for the period between birth and weaning, which is compared between risk factor categories (classes), resulting in an incidence (rate) ratio (IRR), which has a comparable interpretation to that of an Odds Ratio (OR). Clustering of piglets within sows was defined using the survey set command of the statistical package (STATA 12), with sows being defined as random effects both in the univariable and multivariable models.

After running descriptive statistics on categorical outcome variables, their class frequencies were assessed and classes with low frequencies were merged whenever biologically feasible to increase frequencies. For continuous variables, mean and median values as well as standard deviations and confidence intervals were assessed. In the modelling approach they in addition were categorized using quartiles, and the fit of both formats in the model assessed. Potential risk factors were screened for their individual association with the outcome variable using a Poisson regression model with sows as a random effect as described above, and with farm (1, 2, 3) always included as a fixed effect to account for differences between farms. For categorical variables, the different classes (categories) were compared with a predefined baseline category (first class in order) while for continuous variables the average increase of risk resulting from a one-unit change in the risk factor was estimated.

Correlation between different variables, which could affect the final multivariable model, was analysed by screening a Spearman Rank correlation coefficient matrix that included all risk factors considered for the model. A correlation was considered being relevant when r > 0.5. In case of such substantial correlation a decision was taken which of the variables in the pair was to retain for a final model.

Finally, multivariable Poisson regression models were run that included those risk factors identified in the univariable procedure, again with herd as a fixed effect and sow as a random effect. Results of all models were presented as incidence rate ratios (IRR) with related 95% confidence intervals (CI) and corresponding p-values.

In all statistical analyses the level of significance was set at α < 0.05.

## Results

According to the sampling protocol in each herd, sows from three farrowing batches were enrolled leading to a total number of 135 sows and their litters. Due to cross-fostering of piglets from litters not included in the study, 12 litters had to be excluded. Another 11 litters were excluded because their sow was slaughtered before regular weaning. In total, data from 112 litters with 1,127 suckling pigs were the basis for all risk factor analyses, whereas data from the 1,033 nursery pigs that were re-identified at approx. 9 weeks of age only served as basis for some descriptive statistics.

Sows farrowed on average 14.9 piglets per litter (SD: 3.2). The mean number of life born piglets was 13.4 (SD: 2.9) and 10.1 of them were weaned (SD: 1.7), this reflecting a loss of study animals of approximately 24.6%. The reasons for this are comprised by suckling pig mortality but also by cross-fostering of piglets to sows not included in the study. The duration of the suckling period for piglets remaining in the further analysis was 24.8 days on average (mean; SD: 3.9).

### Detection of *M. hyopneumoniae* in sows and piglets by PCR

The detection rate of *M. hyopneumoniae* in sows was 6.3% (7/112) after farrowing and had statistically significant increased to 22.3% (25/112) at the time of weaning (*P* < 0.001).

The prevalence of *M. hyopneumoniae* in suckling pigs was 3.6% (41/1,127) at the time of weaning. There was no association between positive PCR results from nasal swabs obtained from sows after farrowing and positive results from suckling pigs’ nasal swabs sampled at weaning. In contrast, a statistically significant association was found between positivity in sows at the time of weaning and positivity in suckling pigs at the same time using simple chi-square test (*P* = 0.042). Overall 241 piglets were weaned from the 25 PCR-positive sows and *M. hyopneumoniae* was demonstrated in 14 of these pigs (5.8%), whereas the microbe was demonstrated in 27 out of 886 (3.0%) piglets weaned from 87 PCR-negative sows.

Towards the end of the nursery period, when the average age of the pigs was 60.1 days, the prevalence of *M. hyopneumoniae* was 1.2% (12/1033), which was significantly lower than at weaning (*P* < 0.001). The 94 pigs that were not re-tested either had lost their ear tags or had died during the nursery period. On individual pig level, there was no significant association between PCR results at weaning and at the end of the nursery unit (*P* = 0.382).

### Detection of antibodies against *M. hyopneumoniae* in sows and piglets by ELISA

Overall 77.7% (87/112) of all sows were seropositive to *M. hyopneumoniae* 3 weeks ante partum. The average S/P ratio in this group was 0.767 (median; range: 0.403 - 2.660). The 22.3% (25/112) remaining sows considered seronegative demonstrated an average S/P ratio of 0.316 (median; range: 0.131 - 0.385). After farrowing, 53.2% (59/111) of the sows were seropositive with an average S/P ratio of 0.679 (median; range: 0.408 - 3.859). The group of negative sows (46.8%; 52/111) was characterized by a median S/P ratio of 0.296 (range: 0.075 - 0.397). The serum of one sow sampled after farrowing was not available for testing due to severe haemolysis. There was a statistical significant association between seropositivity in sows 3 weeks ante partum and shortly after farrowing (*P* < 0.001).

At 14 days of age, 70.9% (799/1127) of the suckling pigs were seronegative with a median S/P ratio of 0.160 (range: 0–0.392), whereas 29.1% (328/1127) were seropositive. This group showed a median S/P ratio of 0.679 (range: 0.405 - 2.611).

### Risk factor analysis

Categorical data collected from sows and piglets are shown in Tables 2, 3 and 4. Selected continuous data from sows, litters and piglets, which demonstrated a normal distribution, are described in Table 5. The following risk factor analyses are based upon Poisson-type regression models always considering the sow as a random effect.

**Table 2:** Categorical variables and their levels collected from 112 sows

**Variable**	**Level**	**Sows / level**	
		**(n)**	**(%)**
Parity	1. parity	32	28.6
	2. parity	18	16.1
	3. parity	11	9.8
	4. parity	17	15.2
	5. parity	7	6.3
	6. parity	6	5.4
	7. parity	9	8.0
	8. parity	8	7.1
	9. parity	4	3.6
Time of farrowing*	day	66	58.9
	night	46	41.1
Abnormal behaviour (aggression, etc.)	no	105	93.8
	yes	7	6.2
Diseases	no	95	84.8
	local diseases**	5	4.5
	systemic disease incl. PPDS	12	10.7
Time of occurrence of disease	day of partum	1	5.9
	1 day post-partum	8	47.1
	2 days post-partum	2	11.8
	3 days post-partum	4	23.5
	later	2	11.8
Facultative treatment with antibiotics effective against M. hyopneumoniae***	no	94	83.9
	yes	18	16.1
Level of antibodies against *M. hyopneumoniae* ante-partum	S/P ratio < 0,4	25	22.3
	S/P ratio ≥ 0,4	87	77.7
Level of antibodies against *M. hyopneumoniae* post-partum	S/P ratio < 0,4	52	46.8
	S/P ratio ≥ 0,4	59	53.2

*‘day’ linked to working hours, whereas ‘night’ was out of working hours.

**local disease = arthritis, circumscribed dermatitis, shoulder lesion, etc.

***treatments with amoxicillin, ampicillin, colistin and penicillin were considered being NOT effective against *M. hyopneumoniae*, whereas apramycin, enrofloxacin, tetracyclin and tulathromycin were taken into account as being effective against *M. hyopneumoniae.*

**Table 3:** Categorical variables of ‘production data’ and their levels collected from 1,127 suckling pigs

**Variable**	**Level**	**All piglets**		**Mhyo neg.**	**Mhyo pos.**
		**(n)**	**(%)**^**1**^	**n (%)**^**1**^	**n (%)**^**1**^
Gender	Male	572	50.8	554 (51.0)	18 (43.9)
	Female	555	49.2	532 (49.0)	23 (56.1)
Health status after birth	Good	1091	96.8	1051 (96.8)	40 (97.6)
	Moderate/poor	36	3.2	35 (3.2)	1 (2.2)
Special health conditions	None	1026	91.0	990 (91.2)	36 (87.8)
	Congenital conditions	55	4.9	53 (4.9)	2 (4.9)
	Others (e.g. injuries)	46	4.1	43 (3.9)	3 (7.3)
Suckled teat^x^	1^st^ or 2^nd^ pair from cranial	328	29.1	320 (29.5)	8 (19.5)
	3^rd^ or 4^th^ pair from cranial	327	29.0	311 (28.6)	16 (39.0)
	5^th^ to last pair from cranial	181	12.1	173 (15.9)	8 (19.5)
	Varying	291	25.8	282 (26.0)	9 (22.0)
Cross-fostered	No	946	83.9	911 (83.9)	35 (85.4)
	Yes	181	16.1	175 (16.1)	6 (14.6)
Individual contact to new litter mates from other sows due to cross-fostering	No	691	61.3	670 (61.7)	21 (51.2)
	To piglets from negative sows	152	13.5	146 (13.4)	6 (14.6)
	To piglets from positive sows	21	1.9	21 (1.9)	0 (0.0)
	To piglets from sows^ne^	263	23.3	249 (22.9)	14 (34.2)
Individual contact to piglets either negative or positive for *M. hyopneumoniae*	No	385	34.2	385 (35.5)	0 (0.0)
	To foreign negative piglets	368	32.7	368 (33.9)	0 (0.0)
	To foreign positive piglets	36	3.2	36 (3.3)	0 (0.0)
	To positive litter mates	338	30.0	297 (27.3)	41 (100)
Cross-fostering of piglets into	Neighbouring pen	26	14.4	25 (14.3)	1 (16.7)
	Pen in the same compartment	106	58.6	104 (59.4)	2 (33.3)
	Pen in another compartment	49	27.0	46 (26.3)	3 (50.0)
Time of cross-fostering	Day of birth	85	47.0	84 (48.0)	1 (16.7)
	1. day of life	12	6.6	12 (6.9)	0 (0.0)
	2.-7. day of life	46	25.4	43 (24.6)	3 (50.0)
	8.-21. day of life	38	21.0	36 (20.6)	2 (33.3)

Using PCR results from suckling pigs´ nasal swabs as a binomial outcome variable, the incidence rate ratio (IRR; recognizing the first level of each variable as baseline in a univariable Poisson regression analysis) were calculated.

^ne^ not examined for *M. hyopneumoniae.*

^x^based on ≥3 identical results assessed during 5 days of observation.

^1^ column-percent per variable.

**Table 4:** Categorical variables of ‘infection data’ and their levels collected from 1,127 piglets

**Variable**	**Level**	**All piglets**		**Mhyo neg.**	**Mhyo pos.**
		**(n)**	**(%)**^**1**^	**n (%)**^**2**^	**n (%)**^**2**^
Vaccine against *M. hyopneumoniae*	Suvaxyn® M. hyo	386	34.3	361 (33.2)	25 (61.0)
	Ingelvac® M. hyo	441	39.2	427 (39.3)	14 (34.1)**
	Porcilis® M hyo	298	26.5	296 (27.3)	2 (4.9)**
Vaccination against PCV2	No	310	27.5	308 (28.4)	2 (4.9)
	Yes	817	72.5	778 (71.6)	39 (95.1)*
Diseases	No	701	62.2	674 (62.1)	27 (65.9)
	Local diseases	15	1.3	15 (1.4)	0 (0.0)
	Systemic diseases	146	13.0	143 (13.2)	3 (7.3)
	Respiratory diseases	265	23.5	254 (23.4)	11 (26.8)
Routine treatment in the 1^st^ week of life^†^	Amoxicillin	386	34.3	361 (33.2)	25 (61.0)
	Toltrazuril & penicillin	441	39.1	427 (39.3)	14 (34.1)**
	Long-lasting amoxicillin	300	26.6	298 (27.4)	2 (4.9)**
Routine treatment in the 2^nd^ week of life^†^	Long-lasting penicillin	441	66.0	427 (60.7)	14 (93.3)
	Amoxicillin & tulathromycin	277	34.0	276 (39.3)	1 (6.7)**
Routine treatment in the 3^rd^ week of life^†^	Tulathromycin	441	100.0	427 (100)	14 (100)
Any treatment against *M. hyopneumoniae*	No	408	36.2	382 (35.2)	26 (63.4)
	Yes	719	63.8	704 (64.8)	15 (36.6)*
Level of antibodies against *M. hyopneumoniae*	S/P ratio < 0,4	799	70.9	769 (70.8)	30 (73.2)
	S/P ratio ≥ 0,4	328	29.1	317 (29.2)	19 (26.8)

Using PCR results from suckling pigs’ nasal swabs as a binomial outcome variable, the incidence rate ratio (IRR; recognizing the first level of each variable as baseline in a univariable Poisson regression analysis) were calculated. Significant differences are indicated with asterisks.

^†^ if applied.

^1^ column-percent.

^2^ row–percent.

* P-value of IRR <0.05.

** P-value of IRR <0.01.

**Table 5:** Continuous variables and their levels collected from 1,127 piglets

		**All piglets (n = 1,127)**	**Mhyo neg. (n = 1,086)**	**Mhyo pos. (n = 41)**
**Variable**	**Unit**	**Mean /SD/**	**Mean /SD/**	**Mean /SD/**
Length of suckling period	day	24.9 (3.9)	24.8 (3.9)	26.4 (2.7)*
Piglets’ weight at birth	kg	1,4 (0.3)	1,4 (0.3)	1,4 (0.3)
Piglets’ weight at weaning	kg	7,0 (1.8)	7,0 (1.8)	6,9 (1.6)
Daily weight gain	g	225 (57)	225 (57)	207 (57)*
Temperature in the piglet nest at birth^†^	°C	35.0 (4.7)	34.9 (4.4)	34.8 (7.0)
Temperature in the piglet nest at 7 days^†^	°C	33.8 (2.9)	33.8 (2.9)	32.6 (2.5)
Temperature in the piglet nest at 14 days^†^	°C	32.7 (2.8)	32.8 (2.8)	31.8 (1.6)*
Temperature in the piglet nest at 21 days^†^	°C	33.1 (2.5)	33.1 (2.5)	32.5 (1.5)

Using PCR results from suckling pigs’ nasal swabs as a binomial outcome variable, the incidence rate ratio (IRR; recognizing the first quartile within each variable as baseline in a univariable Poisson regression analysis) were calculated. Significant differences are indicated with asterisks.

^†^Figures have been assessed for litter-wise. If at least one piglet of a particular litter was positive for *M. hyopneumoniae* at weaning, then all temperatures of this litter were considered for Mhyo.pos.

* P-value of IRR <0.05.

#### Factors associated with the sow and/or litter

Several factors associated with the sow (e.g. number of parities, time of farrowing, concentration of serum antibodies against *M. hyopneumoniae*, etc.) were suspected to have an impact on the detection rate of *M. hyopneumoniae* in piglets at the end of the suckling period (Table 2). A trend was seen for the PCR-positivity to *M. hyopneumoniae* in the sow. When the individual sow’s nasal swab was positive for *M. hyopneumoniae* at the time of weaning each of her suckling pigs was 1.9 times more likely being also tested positive by PCR compared to piglets from negative sows (*P* = 0.053). Moreover, a significant association between detection of *M. hyopneumoniae* and the number of life-born piglets could be confirmed. In the 31 litters with positive piglets, an average of 13.2 piglets was live born (mean; SD: 2.8), whereas in the remaining litters this number was 13.4 (mean; SD: 3.0). For each additional life-born piglet, the relative change in the incidence rate was 0.9 (*P* = 0.038). None of the other variables determined on sow level was associated with suckling pigs’ positivity to *M. hyopneumoniae* at weaning.

#### Management factors

Routine procedures others than castration of male piglets (grinding of teeth, iron injection, tail docking, etc.) were applied to the 1,127 suckling pigs mainly on the 1st or 2nd day of life (median: 1; range: 0–4).

When teeth grinding was performed, which was the case for 66.3% (747/1127) of the piglets, the IR for *M. hyopneumoniae* infection was 0.3 compared to piglets without teeth grinding (*P* = 0.001). The prevalence among piglets with shortened teeth was 2.8% (20/747), whereas it was 5.5% (21/380) in the group of piglets that has not received this treatment.

After injecting all suckling pigs with 200 mg of iron during the first days of life, 64.2% (723/1127) of the suckling pigs received a second 200 mg injection of iron on the 6th or 7th day of life (median: 7; range: 4–17). The likelihood of detecting *M. hyopneumoniae* at the end of the suckling period in this group was decreased by 70% (IRR: 0.3; *P* = 0.001). Overall 2.1% (15/723) of these piglets treated twice were positive compared to 6.4% (26/404) positives in the group of piglets treated once.

All male piglets were castrated surgically (median: 6; range: 1–17), but this procedure was not associated with the outcome variable, as gender did not (Table 3).

The contact network between piglets as a consequence of cross-fostering was leading to different prevalences of *M. hyopneumoniae* (Table 3). When analysing the quartiles of number of piglets cross-fostered into a litter, no significant effect could be confirmed (IRR: 1.047; *P* = 0.420) apart from a litter effect (*P* = 0.049).

Towards weaning, the likelihood of *M. hyopneumoniae* positivity of piglets’ nasal swabs was increasing by 10% for every day that the suckling period was lasting longer (IRR: 1.1; *P* = 0.011).

#### Environmental factors

The temperatures in the piglet nests, which were measured at specific dates during the suckling period, are presented with the range of their quartiles (Table 6).

**Table 6:** Multivariable poisson regression models identifying risk factors and their influence on the incidence rate ratio

**Variable**	**IRR**	**P-value**	**95% CI**
*Second iron injection in suckling pigs*			
No	1.00	-	-
Yes	0.08	0.08	0.01-1.33
Herd 1 vs. herd 2	5.89	0.22	0.34-101
Herd 1 vs. herd 3	0.67	0.69	0.09-4.94
*Teeth grinding*			
No	1.00	-	-
Yes	**0.10**	<0.01	0.02-0.44
Herd 1 vs. herd 2	**4.76**	0.04	1.08-20.9
Herd 1 vs. herd 3	n.a.		
*Vaccination of suckling pigs against PCV2*			
No	1.00	-	-
Yes	**9.72**	<0.01	2.30-41.0
Herd 1 vs. herd 2	**0.49**	0.03	0.25-0.94
Herd 1 vs. herd 3	n.a.		
*Second routine treatment of suckling pigs*			
No	1.00	-	-
Yes	0.08	0.08	0.01-1.33
Herd 1 vs. herd 2	5.89	0.22	0.34-101
Herd 1 vs. herd 3	0.67	0.69	0.09-4.94
*Number of piglets cross-fostered into a litter*			
1. & 3. Quartile	1.00	-	-
4. Quartile	1.82	0.08	0.94-3.52
Herd 1 vs. herd 2	0.52	0.05	0.27-1.00
Herd 1 vs. herd 3	0.10	<0.01	0.02-0.41
*Temperature in the piglet nest / day of birth*			
1. Quartile (26.5°C-32.2°C)	1.00	-	-
2. Quartile (32.3°C-33.5°C)	0.55	0.17	0.24-1.29
3. Quartile (33.6°C-36.6°C)	0.62	0.27	0.27-1.43
4. Quartile (36.7°C-47.1°C)	**0.40**	0.04	0.16-0.96
Herd 1 vs. herd 2	**0.45**	0.02	0.23-0.89
Herd 1 vs. herd 3	**0.09**	<0.01	0.02-0.37
*Temperature in the piglet nest / 7 day post natum*			
1. Quartile (22.4°C-32.2°C)	1.00	-	-
2. Quartile (32.3°C-33.8°C)	0.47	0.13	0.17-1.25
3. Quartile (33.9°C-35.1°C)	**0.23**	<0.01	0.08-0.63
4. Quartile (35.2°C-42.2°C)	**0.34**	0.01	0.14-0.81
Herd 1 vs. herd 2	0.71	0.39	0.33-1.53
Herd 1 vs. herd 3	**0.11**	<0.01	0.03-0.46
*Temperature in the piglet nest / 14 day post natum*			
1. Quartile (23.6°C-30.9°C)	1.00	-	-
2. Quartile (31.0°C-32.7°C)	1.25	0.54	0.62-2.52
3. Quartile (32.8°C-33.9°C)	0.42	0.10	0.16-1.16
4. Quartile (34.0°C-43.2°C)	**0.26**	0.02	0.09-0.77
Herd 1 vs. herd 2	**0.48**	0.03	0.25-0.94
Herd 1 vs. herd 3	**0.10**	<0.01	0.02-0.42

Using PCR results from suckling pigs’ nasal swabs as a binomial outcome variable, the incidence rate ratio (IRR) were calculated. Significant differences between levels are indicated in bold.

IRR with P-value ≤0.05 are marked in bold.

Inside the farrowing compartments the average temperature measured during the day was 22.5°C (median: 18.0 to 23.6). Highest temperatures on a daily basis ranged from 22.1°C to 27.0°C (median 26.5), and the lower band ranged from 10.8°C to 22.4°C (median 20.0).

The average outside temperature on a daily basis was 7.6°C (median; range −3.0°C to 11.9°C). Highest temperatures per day ranged from 4.1°C to 24.9°C (median 15.9°C) and lowest ranged from −14.8°C to 2.2°C (median −3.2°C).

#### Vaccination

Two different types of vaccines against *M. hyopneumoniae* were used in the study herds: a so-called one-shot vaccine, which is characterized by the fact that it is applied as a single dose only once. In contrast, the two-shot vaccines need at least two injections with 2–4 weeks in-between in order to induce immunity.

In the herd using a one-shot vaccine against *M. hyopneumoniae* in suckling pigs, this was given at 22 days of age (range: 19–30). In the two herds applying a two-shot vaccine to the piglets, the first injection was conducted at approximately 5 to 6 days of age (median: 5; range: 2–23). Subsequently, 58.5% (659/1127) of all suckling pigs in this study received a second dose at 24 days of age (range: 20–43).

Overall 72.5% (817/1127) of all piglets received a vaccine against PCV2. The injection was applied either immediately before or during the weaning process.

#### Antimicrobial treatments

All treatments of sows and piglets with antimicrobials were defined as ‘effective’ or ‘not effective’ against *M. hyopneumoniae* (see Methods).

Sows were treated for various reasons and at varying time (Table 2), but no effect on the outcome variable could be confirmed.

All suckling pigs in this study received a routine injection with antimicrobials ‘not effective against *M. hyopneumoniae*’ within their first days of life (mean: 1; range: 0–3; Table 4). Due to the skewed distribution with 100% in one level no further analysis was possible.

A second injection with antimicrobials was applied to 718 piglets around day 7 (median; range: 1–17; Table 4). This time, 34.0% were injected with a substance ‘effective against *M. hyopneumoniae*’ and they demonstrated a prevalence of *M. hyopneumoniae* of 0.4% at the end of the suckling period. The remaining 66.0% received a substance classified as being ‘not effective against *M. hyopneumoniae*’ and the prevalence of *M. hyopneumoniae* in this group at the time of weaning was 3.2%.

Finally, 441 piglets received a third injection with antimicrobials around day 15 (median; range 7–19), which for them was the first one with a substance ‘effective against *M. hyopneumoniae*’. Again, due to the skewed distribution with 100% in one level no further analysis of this single variable was possible.

Taking all treatments into account, an increasing age whilst receiving the first antimicrobial treatment ‘effective against *M. hyopneumoniae*’ was associated with an increase of the IR of *M. hyopneumoniae* infection of 20% per day (IRR: 1.2; *P* = 0.004).

#### Multivariable risk factors analysis

Poisson regression models were used to identify the impact of single risk factors on the outcome variable while considering the herd as a fixed effect and the sow as a random (study design) effect. In four variables - ‘*Teeth grinding’, ‘Temperature in the piglet nest at birth’, ‘Temperature in the piglet nest 7 days post natum’ and ‘Temperature in the piglet nest 14 days post natum’* - levels leading to an IRR < 1 could be identified, this reflecting a protective effect regarding suckling pigs’ positivity to *M. hyopneumoniae* at the time of weaning. Noteworthy, in all these variables a significant herd effect (*P* < 0.05) was observed. The vaccination of suckling pigs against PCV2 was linked to an IRR > 1 and, therefore, a risk factor for the infection of piglets with *M. hyopneumoniae* at the end of the suckling period. This risk factor was also affected by a significant herd effect.

## Discussion

Suckling pigs infected with *M. hyopneumoniae* are considered as initiators for the spread of *M. hyopneumoniae* infections during the nursery and fattening period [4,5,14]. It is hypothesised that identification of risk factors can help to create intervention strategies against a frequent transmission of *M. hyopneumoniae* from sows to their offspring and thereby further transmission to pen mates in the nursery and growing units. However, many studies investigating potential risk factors for *M. hyopneumoniae* infections in pigs were predominantly focused on weaned pigs, e.g. growing and finishing pigs [10,18-21] and did not highlight individual risk factors, which increase the probability of detecting *M. hyopneumoniae* in suckling pigs. In the present study the occurrence of *M. hyopneumoniae* infections in suckling and nursery pigs was investigated and various factors potentially influencing the infection status of piglets at the end of the suckling period were analysed.

### Detection of *M. hyopneumoniae* in sows by PCR

An increased positivity for *M. hyopneumoniae* of the sows’ nasal mucosa was expected around the time of birth. It was hypothesised that a stressful situation as giving birth to piglets would impact the immune system of the sows. Interestingly, the findings of the present study are in contrast to this hypothesis and a previous report, where a decrease in the prevalence from farrowing to weaning was observed at least in some groups [22]. Potentially, the present, ‘delayed’ outcome was due to the slow replication / growth of *M. hyopneumoniae*, and the loss of antibodies shortly before the birth [23] could have affected the course of this increase of detection rates.

### Detection of *M. hyopneumoniae* in suckling pigs by PCR

The prevalence 3.6% is low compared to other reports, where higher rates of 7.7% [22] and 11.3% [13] were found. However, herds examined in these studies had been selected by either frequent detection of *M. hyopneumoniae* in other age groups combined with occurrence of enzootic pneumonia or by severe clinical symptoms of respiratory disease in the group of question, i.e. among the suckling pigs. In contrast, herds in the present study were enrolled, when only few suckling pigs were tested positive for *M. hyopneumoniae*. Noteworthy, in one study describing the course of infection from birth to slaughter, a well comparable detection rate of 3.8% was found in suckling pigs at the age of weaning [14]. Moreover, a recent randomized cross-sectional study reported an overall detection rate of 3.9% in this particular age group [24]. Notwithstanding, the detection rate assessed with PCR on nasal swabs can be influenced by the virulence of the strain (i.e. course of infection) and the imperfect sampling site [25], and it should be considered that testing 20 suckling pigs per herd results in a maximum possible prevalence of approximately 13%, which - by chance - remains undetected (assumed population size: 100; level of confidence: 95%).

The link between sows colonisation status and positivity in suckling pigs at weaning as found in the present study was already assumed by others [22], and complies to within-herd transmission pathways described for *M. hyopneumoniae*[26].

### Detection of *M. hyopneumoniae* in nursery pigs by PCR

The decreased detection rate of 1.2% among nursery pigs is in accordance to the reproduction ratio of *M. hyopneumoniae* infection during 6 weeks of nursery, which has been estimated being R_0_ = 0.56 for unvaccinated and R_0_ = 0.71 for vaccinated nursery pigs [27]. Another study reported an average R_0_ = 1.16 during a 6 week nursery period [4], but this was elaborated in an experimental set-up with inoculation of seeder pigs. It was shown that high virulent strains of *M. hyopneumoniae* lead to higher reproduction ratios (R_0_ > 1) than low virulent strains (R_0_ < 1).

### Detection of antibodies against *M. hyopneumoniae* in sows and suckling pigs by ELISA

Sows have been tested for antibodies against *M. hyopneumoniae* three weeks prior to and shortly after farrowing in order to determine the transfer of maternal antibodies to the progeny. It is known that a decrease of the concentration of serum antibodies identified by lower S/P ratios or equivalent values over time, as well as a decrease in the overall prevalence of ‘positives’ during this pre-farrowing period is due to a transfer of serum antibodies into the colostrum [28,29]. In the present study, neither the S/P ratios three weeks prior to farrowing, the S/P rations shortly after farrowing nor the differences (data not shown) were associated with the detection of *M. hyopneumoniae* by PCR in nasal swabs from suckling pigs. A similar observation was already made in a previous study [22].

The serological status of the suckling pigs at 14 days of age also did not show any impact on the prevalence of *M. hyopneumoniae* suckling pigs at the time of weaning. Former studies have shown that high levels of maternally derived antibodies facilitate prevention of *M. hyopneumoniae*-infection of sucking pigs [30,31], but this effect could neither be confirmed in the present study nor in others [3,27].

### Risk factors

An increase in the number of life born piglets per litter was linked to a lower incidence rate of *M. hyopneumoniae* in suckling pigs at weaning. Even though it was not expected that reproductive performance has an impact on *M. hyopneumoniae*-infections, there is no doubt about the outcome, since an equal finding has been reported recently in the same context [32]. These findings may indicate that high performing herds truly apply extensive hygiene measures and excellent animal care taking, which however were not captured in the studies.

Another observation with regard to management was that grinding piglets’ teeth was leading to a significant lower incidence rate of *M. hyopneumoniae* infections. Whether this effect was confounded by an increased colostrum uptake or a higher daily weight gain due to better milk supply by the sow in accordingly treated litters could neither be confirmed nor ruled out. Nonetheless, ‘teeth grinding’ was one of the few variables remaining in the final multivariable Poission regression model with significant impact on positivity to *M. hyopneumoniae*.

The application of a second dose of iron was associated with a decrease of the incidence rate. Because of the high growth rate of piglets and low iron content of the sows’ milk, conventionally raised suckling pigs usually need additional iron during their first week of life in order to prevent anaemia [33], and it is possible that some piglets, only receiving 200 mg iron shortly after birth, are borderline anaemic when weaned at day 25 or later. Since it has been shown that increasing the iron supply influences several parameters including immunity [34], this second iron injection might have prevent piglets also from infection with *M. hyopneumoniae*.

With an increase of the duration of the suckling period piglets were more often positive to *M. hyopneumoniae* at weaning. Considering that the transmission of *M. hyopneumoniae* from sows to their offspring is likely depending on duration of exposure, these results are fairly conclusive. Similar findings have been reported recently [32]. It remains unclear, whether the ‘lower average daily weight gain’ or the increased length of the suckling period, which is an inevitable consequence of the first one, is responsible for the observed effect. Farmers often postpone the weaning date, when piglets are not heavy enough. Unfortunately, there is no way to sort this out, so that further research on this topic is highly recommended.

The floor temperature in the piglet nest demonstrated a significant association with the differences in the incidence rates. Noteworthy, the temperatures as presented in Table 6 do neither consider piglets’ behaviour nor their location in the farrowing pen. Obviously, piglets were not lying in the nest area, when temperatures reached more than 45°C, which was due to a failure of the electrical heating device. The significant impact of piglet nests’ temperatures were also confirmed in the final multivariable model. These findings were in accordance to previous reports describing the interactions between environmental conditions and development of the pig’s immune system. It has been shown that low environmental temperatures aid a reduced suckling activity and a delayed development of immune-competence [35-37].

Vaccination of suckling pigs against PCV2 was linked to an IR of 9.7, but the vaccination, the weaning and the sampling were mainly performed in parallel or in very brief sequences, this ruling out a direct influence of the vaccine. Reasons for an association are more likely respiratory disease in older pigs of the same herd, which demand for vaccination against potential initiators like PCV2. In this context, the high frequencies of co-infections with PCV2 and *M. hyopneumoniae* and the potentiating effect on clinical symptoms have to be considered [38,39].

In this observational study, sows and suckling pigs have been treated due to the occurrence of diseases and for metaphylactic reasons, respectively, with various antimicrobials at different points in time. The authors of this article were neither responsible for the application of mass treatment nor have been asked for their opinion. Instead, all treatments had been advised by the herd attending veterinarians and were based on particular disease histories in the herds. It should be noted that attempts of preventing *M. hyopneumoniae* infections in pigs solely by antibiotic treatment is usually not sustainable and is not accounting for the veterinarians’ responsibility for prudent use of antimicrobials, prevention of bacterial resistance and general public health issues!

Treatments of suckling pigs in two farms included an injection of tulathromycin, which is known to be highly effective against *M. hyopneumoniae*[40]. Due to the fact that a relatively low prevalence of *M. hyopneumoniae* in suckling pigs at weaning was observed in the one farm compared to the other, an effect of time of applying antimicrobials could be discussed. However, this does not prove causality and the unequal distribution of positive suckling pigs among the three study herds could likely have biased this outcome. Even though pathogen elimination by applying antimicrobials to suckling pigs has been used to develop the procedure of ‘medicated early weaning’ [41,42], there are serious concerns regarding this mass treatment: An elimination of *M. hyopneumoniae* in growing pigs by applying antimicrobials once or even twice during the suckling period should not be expected. Moreover, the use of antimicrobials for metaphylaxis is contrary to recent demands on prudent use of drugs in both human and veterinary medicine.

## Conclusions

Several individual risk factors being associated with the detection of *M. hyopneumoniae* in suckling pigs at the age of weaning have been identified. Since single infected piglets are supposed to act as initiators for the transmission of this pathogen in nursery and fattening pigs, the elimination of the risk factors described in the present study may help to reduce the incidence rate of *M. hyopneumoniae* and thereby lower the probability of high prevalences in older pigs. It was shown that excellent management including animal care is very useful in preventing *M. hyopneumoniae* infections in suckling pigs, which ideally should be accomplished by excellent housing of sows and their piglets.

## Competing interests

The authors have no financial or personal relationship with people or organisations that could inappropriately influence or bias the content of this paper. The sponsor, who was involved in the development of the study concept, worked neither on other study issues nor on the content of this manuscript.

## Authors’ contributions

EGB, MGD and HN designed the cross-sectional study and SD and HW carried out the farm visits and data collection. SD and HW performed the laboratory tests under supervision of HN. ASF assisted with statistical analysis and MGD performed statistical analysis. HN drafted the manuscript. ASF, MGD and EGB critically revised the manuscript and all authors read and approved the final manuscript.
